# SIR+ models: accounting for interaction-dependent disease susceptibility in the planning of public health interventions

**DOI:** 10.1038/s41598-024-63008-9

**Published:** 2024-06-05

**Authors:** Maria M. Martignoni, Aura Raulo, Omer Linkovski, Oren Kolodny

**Affiliations:** 1 Department of Ecology, Evolution and Behavior, Faculty of Sciences, A. Silberman Institute of Life Sciences, Hebrew University of Jerusalem, Jerusalem, Israel; 2 Department of Biology, University of Oxford, Oxford, UK; 3 Department of Computing, University of Turku, Turku, Finland; 4 Department of Psychology and The Gonda Multidisciplinary Brain Research Center, Bar-Ilan University, Ramat-Gan, Israel

**Keywords:** Microbiome, Public health, SIR, SIR+, Mathematical model, Social distancing, Physical distancing, Optimal policy, Microbiome transmission, Disease spread, Disease management, Health policies, Immunity debt, Applied mathematics, Health policy

## Abstract

Avoiding physical contact is regarded as one of the safest and most advisable strategies to follow to reduce pathogen spread. The flip side of this approach is that a lack of social interactions may negatively affect other dimensions of health, like induction of immunosuppressive anxiety and depression or preventing interactions of importance with a diversity of microbes, which may be necessary to train our immune system or to maintain its normal levels of activity. These may in turn negatively affect a population’s susceptibility to infection and the incidence of severe disease. We suggest that future pandemic modelling may benefit from relying on ‘SIR+ models’: epidemiological models extended to account for the benefits of social interactions that affect immune resilience. We develop an SIR+ model and discuss which specific interventions may be more effective in balancing the trade-off between minimizing pathogen spread and maximizing other interaction-dependent health benefits. Our SIR+ model reflects the idea that health is not just the mere absence of disease, but rather a state of physical, mental and social well-being that can also be dependent on the same social connections that allow pathogen spread, and the modelling of public health interventions for future pandemics should account for this multidimensionality.

## Introduction

Physical distancing (often called ‘spatial’ or ‘social distancing’,^1,2^) has been implemented around the world as a non-pharmaceutical intervention to control pathogenic spread. Particularly during the COVID-19 pandemic, physical distancing policies have efficiently contributed to slowing the spread of the infection when time was needed to strengthen public health preparedness, and have overall tremendously helped prevent a large number of infections and avoid healthcare burden^3,4^. On the other hand, prolonged physical distancing has also negatively impacted other dimensions of health, leading to an increase in mental health issues^5–7^, substance use^8–10^, domestic violence^11–13^, and children’s social care^14,15^, besides causing a reduction in exposure to a wide diversity of microbes^16–18^, with consequences at individual and societal levels that we are just beginning to understand^13,19–23^. For instance, microbial exposure may be critical to training our immune system or maintaining its normal level of activity^24–26^. Consistently reduced exposure to microbial diversity has also been linked to the insurgence of auto-immune diseases^27–30^, such as inflammatory bowel disease^31^, allergies and asthma^32^, atopic-dermatitis^33^, and even to depression and anxiety^34^. Furthermore, decreased human interactions may lead to changes in circadian clocks and dietary habits, and promote physical inactivity, with extensive negative health impacts^35–41^.

The positive effects of physical distancing, such as the number of averted infections, have been easy to appreciate, thanks to the development of mathematical models that could be used to simulate epidemic spread if physical distancing had not been in place^42,43^. The negative effects of physical distancing, however, have been hard to grasp, mainly because these negative consequences are often to be realized in the future. Only toward the end of the COVID-19 pandemic researchers managed to gather data and develop methodologies to help unveil the multidimensional impact of physical distancing on a population’s health and social functioning^13–15,18,22,44–46^. Perhaps due to this proficiency in predicting the positive effects of physical distancing on decreasing infection risk, and the lack of data and mathematical models required to delineate its negative health effects, public health policies implemented during the pandemic have tended to follow recommendations based on the modelling of positive effects of physical distancing, while its negative consequences have been appreciated to a limited extent.

Increasing physical distancing might not always cause a reduction in infection risk, as often assumed in classic epidemiological models. Reduced human-to-human interactions may impair a population’s ability to cope with the infection and aggravate its effects, suggesting that in some cases an intermediate extent of social distancing may be the optimal balance between these opposing considerations. Particularly in already vulnerable populations, such as those facing poverty and food insecurity from the economic effects of the pandemic response, physical distancing can create a situation in which people are less fit, eat less healthily, sleep less, have depleted microbiome exposure, and are more likely to suffer from social isolation, loneliness, anxiety, or depression^17,36,38,47,48^. These changes may compromise immune functioning, affect a population’s susceptibility to infections, and increase the risk of developing severe illness^47,49–55^. Thus, if on one side physical distancing may decrease pathogen transmission, on the other side it may lead to a loss of benefits of social living affecting our ability to resist infection and tolerate disease.

Here, we present a modelling approach that we hope will trigger a discussion regarding how the possible health consequences of a lack of interactions can be modelled alongside pathogenic transmission for the evaluation of public health interventions. For this purpose, we develop what we call an SIR+ model, i.e., a ‘Susceptible–Infected–Recovered’ mathematical framework that considers positive as well as negative effects of physical distancing on infection risk. As in classical SIR models, a low contact rate decreases the rate of encounter between susceptible and infected individuals, and therefore the rate of pathogen transmission. However, in our model a low contact rate may also decrease other interaction-dependent health benefits which affect a population’s ability to resist and tolerate pathogens. We discuss scenarios in which interaction-dependent health benefits may lower, or even offset, infection risk. Additionally we consider, in light of the particular costs and benefits that physical distancing may have, which interventions may provide maximal health-benefits of social interactions while minimizing pathogen spread.

Our work provides a new way to think of epidemiological models in which infection and hospitalization rates are not necessarily steadily decreasing when contact is minimized, but show complex responses to distancing. We hope that our work will inspire new experimental testing, and start a discussion on how current epidemiological models may be extended to consider the impact on health of multiple interaction-dependent factors in the planning of public health interventions.

## Model and methods

### The SIR+ model

We consider a classic SIR compartmental model^56^ in which a population is divided into susceptible individuals, infected individuals, and individuals who have recovered from infection, whereby the variables *S*, *I* and *R* represent the fraction of the population found in each compartment. Individuals can move from the *S* to the *I* compartment by acquiring the infection through contact at rate $\beta$, representing the average number of susceptible individuals infected by one infections individual, per contact and per unit time. Infected individuals recover and move to the *R* compartment, at rate $\gamma$.

We consider that interaction-dependent health benefits *H* are acquired through contact between and among any pair of individuals in the *S*, *I* and *R* compartments, at rate $\zeta _{(H)}$, where the dependency of $\zeta _{(H)}$ on *H* is explained in Section “Processes driving changes interaction-dependent health benefits”. We consider that under certain circumstances contact with infected or recovered individuals may lead to a different contribution to the acquisition of interaction-dependent health benefits than contact with susceptible individuals, as quantified by fractions $p_I$ and $p_r$. Variable *H* can represent direct or indirect health benefits acquired through human-to-human interaction which affect immune resilience. Direct health benefits include psychological well-being and reduced stress physiology, including levels of stress, depression, hormonal responses (e.g., the production of oxytocin), and stress physiology anomalies, such as stress hyper- and hyporeactivity common in different forms of depression. Indirect health benefits include increased microbiota exposure, microbiome functionality, or improved diet and physical activity, all of which may affect colonization resistance and immune system training and activation. In the absence of contact, interaction-dependent health benefits *H* are lost at rate $\delta$.

The above-described dynamics can be represented by the following system of differential equations:

1a $$\begin{aligned} \frac{d H}{dt}&= \zeta _{(H)} (S+ p_I I+ p_R R)^2 -\delta H \, , \end{aligned}$$

1b $$\begin{aligned} \frac{d S}{dt}&= -{\beta _{(H)} I S } \, , \end{aligned}$$

1c $$\begin{aligned} \frac{d I}{dt}&= {\beta _{(H)} I S } - \gamma I \, , \end{aligned}$$

1d $$\begin{aligned} \frac{d R}{dt}&= \gamma I\, . \end{aligned}$$

Note that Eqs. (1b,c,d) represent the classic SIR model, where the ratio $\beta /\gamma$ can be defined as the basic reproduction number $R_0$, where an epidemic in an initially fully susceptible population occurs only for $R_0 = \beta /\gamma > 1$. In our model, however, the acquisition of interaction-dependent health benefits may affect the ability to *resist* infection (and thus affect the transmission rate $\beta$, see section “Processes determining infection transmission and disease severity”), or the ability to *tolerate* infection (and thus affect the probability of experiencing severe symptoms given infection *p*, see section “Processes determining infection transmission and disease severity”). We call our model an ‘SIR+ model’, as it accounts for the transmission of pathogenic agents causing infection, as well as for other interaction-dependent factors that can affect infection resistance and disease tolerance.

**Figure 1:**
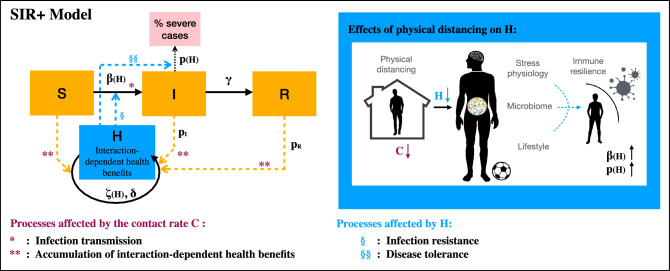
A schematic representation of the SIR+ model of Eq. (1). Like in a classical SIR model, susceptible individuals become infected at rate $\beta$ and successively recover at rate $\gamma$, where variables *S*, *I*, and *R* represent the fraction of the population in the susceptible, infected or recovered compartments at a particular time. Interaction-dependent health benefits (*H*) are acquired through contact between any group of individuals in the population (namely between *S* and *S*, between *S* and *I*, between *I* and *R*, etc.) at rate $\zeta (H)$ (Eq. 2). Interaction with infected and recovered individuals may lead to a different contribution to *H*, as quantified by parameters $p_I$ and $p_R$. We consider that *H* affects (§) infection resistance, and thus the ability of a pathogen to transmit from one individual to the other (parameter $\beta (H)$), or (§§) disease tolerance, and the probability of experiencing severe illness from infection (parameter *p*(*H*)). Physical distancing can lead to a loss of interaction-dependent health benefits and to increased stress physiology, decreased microbial exposure, and unhealthy lifestyle choices, overall affecting immune resilience and a population’s ability to resist infection and tolerate disease. In our model, the contact rate *C* determines (*) the value of the transmission rate $\beta (H)=C \alpha (H)$, by affecting the rate of contact between susceptible and infected individuals, and (**) the acquisition of interaction-dependent health benefits *H*, by affecting the rate of contact between any individuals.

A schematic representation of our SIR+ model is provided in Fig. 1. Default parameter values are provided in Table A.1. Parameter values driving the SIR dynamics are loosely based on SARS-CoV-2 epidemiology, while other parameters are arbitrary, as so far links between increased interactions and decreased susceptibility to infections have only been established qualitatively. Our purpose is therefore to highlight possible scenarios which may occur for a wide range of parameters.

In what follows, we outline the empirical observations that motivate the formulation of our SIR+ model and how they may be implemented in the general framework proposed above. We first discuss the processes determining the acquisition and loss of interaction-dependent health benefits (section “Processes driving changes interaction-dependent health benefits”). We then discuss how interaction-dependent health benefit can affect infection transmission and disease severity (section “Processes determining infection transmission and disease severity”). In section “The role of the contact rate *C* and scenarios of interest”, we outline how our framework can be used to investigate three scenarios of interest (i–iii). The resulting dynamics emerging from these scenarios are presented in section “Results”, and discussed in section “Discussion”. All the code developed for the manuscript is publicly available on GitHub, at https://github.com/nanomaria/SIRplus.

### Processes driving changes interaction-dependent health benefits

#### Acquisition of interaction-dependent health benefits

The dynamics of acquisition of interaction-dependent health benefits is regulated by Eq. (1a), and by the value of $\zeta (H)$. It is reasonable to consider that the acquisition of interaction-dependent health benefits should increase through human contact till a certain maximum $H_{max}$ is reached, above which increasing contact does not further contribute to an increase in health benefits. When *H* is smaller than the possible acquirable benefits $H_{max}$, the rate $\zeta (H)$ at which health benefits are acquired through contact assumes a positive value which depends on the average number of contacts of each individual per day (that is, on the contact rate *C*), on the maximal possible contribution to *H* of each contact (parameter *h*), and on the fraction of contacts *k* that effectively provides this contribution (parameter *k*, ranging from 0 to 1). When *H* is greater than $H_{max}$, contact no longer contributes to an increase in *H*. We write:

2 $$\begin{aligned} \zeta _{{(H)}} = \left\{ \begin{array}{lll} C\,h \,k &{}\quad \textrm{for} &{}\quad \mathrm {H~\le H_{max}} ,\\ 0 &{}\quad \textrm{for}&{}\quad \mathrm {H~> H_{max}} . \end{array} \right. \end{aligned}$$

Note that $\zeta (H)$ increases with increasing *C*, *h*, and *k* till the maximum $H_{max}$ is reached (see SI C, Fig. C.1). The fractional contribution of each contact to the acquisition of interaction-dependent health benefits is indicated by parameter *k*, and may range from 0 to 1. When $k \rightarrow 1$, each contact leads to a maximal contribution to *H*, while when $k \rightarrow 0$ interaction with multiple contacts is required for the acquisition of the same health benefits. A small *k* can represent the situation in which physical interactions are not particularly favourable for psychological well-being or do not provide exposure to essential microbes, despite leading to pathogen spread. Parameter *k* may also represent the distinctness of microbiomes in one’s contacts. When interacting with other people with highly similar microbiomes to one’s own, exposure to microbes carried by others is not as enriching and diversifying as when interacting with people with microbiomes highly different to one’s own, as the latter group hosts more taxa that do not already exist in one’s microbiome. Thus, a population with a high average microbiome similarity may be considered an example of a case of low *k*, while a population with a low average microbiome similarity would be a case of high *k*.

In this manuscript we present a SIR+ model in its most basic form. Therefore, we chose to focus on the positive effects of interactions, such that contact can only lead to an increase in interaction-dependent health benefits *H*. In some circumstances, however, the positive effects of contact may co-occur with additional, often neglected, negative effects of interactions, such as the risk of re-infection, or the spread of parasites or new pathogenic agents^57,58^. Future versions of our SIR+ model may account for these effects, as well as for additional compartments and epidemiological features, such as the presence of pre-symptomatic or quarantined individuals, contact tracing, or population demographic and heterogeneity^58–60^.

#### Loss of interaction-dependent health benefits

Although it is not always easy to distinguish a causal relationship between lack of contacts and changes in microbial diversity, or in the release of stress hormones, it is undoubted that social isolation can negatively affect psychological well-being and microbial exposure. Social isolation tends to lead to immunosuppressive stress and depression, as shown by experimental research on rodents^61,62^ and by several COVID-19 studies^63,64^. Some evidence also suggests that social contact can be important for the maintenance of the resident microbiome, and that social structure can affect microbiome composition in several ways, where microbiome diversity tends to be higher in more social individuals^65–71^. Finally, in some cases, social isolation may affect both microbiome composition and the production of stress hormones^72–75^. Thus, a decrease in human-to-human contact during the COVID-19 pandemic, together with extensive use of hand sanitisers, changes in dietary habits, and viral infection, may have led to losses in microbiome richness and diversity^20,22,23,76–79^.

In our model we account for these effects by considering that, in the absence of contact, interaction-dependent health benefits are lost in a population at rate $\delta$ (see Eq. 1a). The consequences of this loss for immune resilience are discussed in section “Processes determining infection transmission and disease severity”. In SI C, we analyse Eqs. (1a) and 2, and discuss how the balance between the acquisition (determined by parameters *C*, *k*, and *h*) and loss of interaction-dependent health benefits (determined by parameter $\delta$) regulates the interaction-dependent health benefits of a population at equilibrium, and how fast an equilibrium value is reached.

### Processes determining infection transmission and disease severity

As discussed above, social isolation can affect stress physiology and microbiota exposure. These changes may influence immune resilience, and our ability to resist infection and tolerate disease, where with infection ‘resistance’, we refer to the ability of a host to prevent or reduce pathogen load, while with disease ‘tolerance’ we refer to the ability of a host to limit the health impact of a given pathogen^80^.

For instance, diversity of microbial exposure may be essential to guide immune maturation, shape immune responses, such as providing the ability to distinguish pathogens from microbial allies, and maintain the immune system at its normal levels of activity^24,81–86^. Thus, physical contact may be necessary to enhance personal immune response capability, as recently reviewed in Sarkar et al.^18^. This can occur through the transmission of specific taxa, or as an emergent property of the whole microbiome.

Mechanisms of passive or active social immunization through the transmission of specific taxa can occur through the spread of mild pathogens that stimulate the immune system, or through the passive transfer of protective immune effectors^87–90^. Social immunization through microbial exposure is well documented for insect colonies^91–96^, and for vertebrates^97–101^, and their significance in human societies has also been repeatedly discussed^25,102–106^.

Having a diverse microbiome per se can also provide protection from disease^107–110^, where protection against infection and severe disease can be considered as an emerging property of the whole microbiome. A diverse microbiota community can be more successful in outcompeting pathogen invaders^111^, and in defending the host against pathogenic infections^86,112,113^. Although significant debate remains about how microbiota diversity relates to susceptibility to infection, to the incidence of severe disease, and further to health^114^, a link between microbiome diversity and individual susceptibility has been reported for some diseases. For example, microbiome dysfunction has been shown to increase susceptibility to COVID-19 infection, e.g. by affecting viral invasion and replication and providing thereby infection resistance^115,116^, as well as decreased tolerance to disease severity, e.g., by influencing the recruitment of immune cells, or by influencing gut microbiota composition^117–119^. Additionally, social isolation can affect the production of stress hormones (as discussed in section “Processes driving changes interaction-dependent health benefits”), that can suppress immune function directly^120–126^, or indirectly, by affecting microbiome composition^75,127,128^.

We discuss how these considerations can be taken into account in our framework, to explore the consequences of physical distancing on a population’s resistance to infection (section “Infection resistance and the *α*(*H*) function”) and/or tolerance against disease (section “Disease tolerance and the *p*(*H*) function”).

#### Infection resistance and the $\alpha (H)$ function

**Figure 2:**
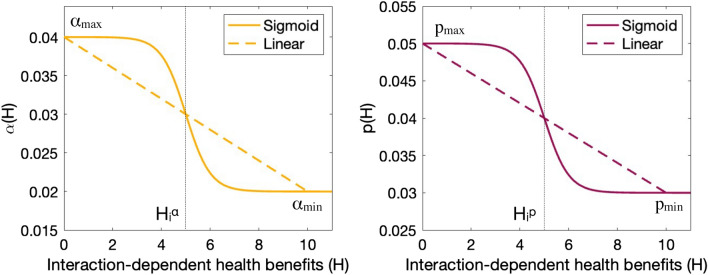
(**a**) Probability of infection transmission given a contact (function $\alpha (H)$, Eqs. 3 and 4), and (**b**) probability of experiencing severe illness from infection (function *p*(*H*), Eqs. (6) and (7)), as a function of increasing interaction-dependent health benefits *H*.

The infection transmission rate $\beta$ depends on the average number of contacts of each individual per day (given by *C*) and on the probability of contracting infection given a contact (given by $\alpha$), with $\beta = C \alpha$. To model a change in the ability of a population to resist infection, we consider that $\alpha$ is lower when interaction-dependent health benefits *H* are large. Under these conditions, the infection transmission rate $\beta (H)=C\alpha (H)$ can be described as a function of *H*. As interaction-dependent health benefits increase, the probability of infection given a contact (i.e., function $\alpha (H)$) decreases from $\alpha _{max}$ to $\alpha _{min}$ and the population becomes more resistant to disease, as shown in Fig. 2a. We consider a situation in which the decrease from $\alpha _{max}$ to $\alpha _{min}$ occurs linearly with increasing *H*, i.e.,

3 $$\begin{aligned} \alpha (H) = \left\{ \begin{array}{lll} - \dfrac{(\alpha _{max} - \alpha _{min})}{2 \, H_i^{\alpha }} H + \alpha _{max}&{}\quad \textrm{for} &{}\quad H \le 2\, H_i^{\alpha },\\ \alpha _{min}&{}\quad \textrm{for}&{}\quad H>2\,H_i^{\alpha }. \end{array} \right. \end{aligned}$$

We also consider the case in $\alpha _{max}$ changes to $\alpha _{min}$ as *H* approaches $H_i^{\alpha }$, and $\alpha (H)$ can be modeled as a sigmoid function, i.e.,

4 $$\begin{aligned} \alpha (H) = \alpha _{{min}} + \frac{\alpha _{max} - \alpha _{{min}}}{2} [ 1- \tanh (H- H_i^{\alpha })]. \end{aligned}$$

Parameter $H_i^{\alpha }$ represents the inflection point of the sigmoid. A linear decrease in $\alpha$ as a function of *H* (Eq. (3) and Fig. 2a, scattered line), represents the case for which each interaction equally contributes to increased pathogen resistance till the maximal acquirable benefit is reached. A sharp decrease from $\alpha _{max}$ to $\alpha _{min}$ occurring at $H \simeq H_i^{\alpha }$ (Eq. (4) and Fig. 2a, solid line) represents the situation in which infection resistance arises and is maintained as long as *H* is greater than a certain threshold value. .

#### Disease tolerance and the *p*(*H*) function

Interaction-dependent factors can affect not only infection transmission, but also the probability of developing severe illness from the infection. To illustrate this possibility, we consider that a higher proportion of the infected population suffers from severe illness when interaction-dependent health benefits are low. We model this proportion by considering that infected cases *I* can be divided into non-severe ($I_{ns}$), and severe cases ($I_s$), with $I = I_{ns} + I_{s}$. The dynamics of populations $I_{ns}$ and $I_{s}$ is given by:

5a $$\begin{aligned} \dfrac{dI_{ns}}{dt} =&(1-p(H)) \beta S I - \gamma I_{ns} \, , \end{aligned}$$

5b $$\begin{aligned} \dfrac{dI_{s}}{dt} =&p(H) \beta S I - \eta I_{s}\,, \end{aligned}$$

with $\eta$ being the recovery time from severe infection.

Thus, the proportion of infected people suffering from severe illness *p* can be modelled as a function of *H*, with $p_{max}$ and $p_{min}$ corresponding to the maximal and minimal proportion of new infections developing into severe cases. Function *p*(*H*) can be modelled similarly to $\alpha (H)$, and decreases from $p_{max}$ to $p_{min}$ as *H* increases (see Fig. 2b). As for $\alpha (H)$, the function *p*(*H*) can be represented as a linear or sigmoid function of *H* (see SI B). If infection resistance is unaffected by *H*, we consider that $\beta = C \alpha _{max}$. Otherwise, $\beta = C \alpha (H)$.

### The role of the contact rate *C* and scenarios of interest

As explained in section “The SIR+ model” and illustrated in Fig. 1, in the model two processes are initiated through contact: (*) The transmission of pathogen agents, contributing to infection spread, and (**) an increase in interaction-dependent factors that may benefit health directly (e.g. by improving a population’s psychological well-being) or indirectly (e.g. by increasing exposure to a wide diversity of microbes that are essential to our health). We consider the case in which a population can acquire through contact the health benefits necessary to increase resistance to infection or disease tolerance (a situation which occurs for $H_i^{\alpha },H_i^p< H_{max}$), and we explore how physical distancing, modelled as variation in the contact rate *C*, may affect the spread of an infection in a population when different interaction-dependent factors are taken into account. Specifically, we will discuss propositions (i)–(iii), described below.

**i. Under which circumstances may physical distancing increase infection risk?**

We investigate the circumstances under which physical distancing, which in our simulations is implemented through a decrease in the contact rate *C*, may increase infection transmission and the incidence of severe illness, due to the loss of interaction-dependent health benefits. We consider that an epidemic outbreak is experienced by a population after the contact rate *C* has been kept constant for a long time (i.e., *H* has already reached an equilibrium value $H^*$ corresponding to a specific value of *C*, see Eq. 9). To investigate changes in infection resistance we compute, for each value of *C*, the basic reproduction number $R_0 = \beta (H)/\gamma$ (with $\beta (H)=C\alpha (H)$). For each $R_0$, we compute then the corresponding epidemic peak, that is the proportion of the population infected when the epidemic curve reaches its maximal height. To investigate changes in disease tolerance, we compute for each value of *C* the peak value in the proportion of severe cases, that is, the maximal value assumed by $I_s$ when the probability of experiencing severe illness *p*(*H*) depends on *H* (see Eq. 5b).

**ii. How do differences in the contribution of contact to the acquisition of interaction-dependent health benefits affect the impact of physical distancing?**

We repeat the procedure described in (i) by considering different values of *k* (namely $k=0.25, 0.5, 0.75$ and 1), representing the fraction of contacts that contribute to the acquisition of interaction-dependent health benefits.

**iii. What are the possible consequences of the abrupt relaxation of physical distancing on the spread of future epidemics?**

While in propositions (i) and (ii) we consider a single outbreak of a single pathogen, occurring within a relatively short time scale, in (iii) we are looking at the situation in which physical distancing has been in place for a long time, such that interaction-dependent health benefits have been lost and the population is now more susceptible to infection and/or less tolerant to disease. The spread a of a new pathogen in this population can therefore lead to more infections and more severe cases than in a population that did not experience social isolation. In our simulations, we consider that the contact rate *C* has been held constant to a low value ($C=5$) for which *H* has reached its equilibrium value $H^*$ (Eq. 9). Physical distancing measures are then released ($C=10$) and a new pathogen starts spreading in the community while interaction-dependent health benefits are low. We compare the occurring epidemic dynamics with what could otherwise be observed if physical distancing had not led to changes in *H* (i.e., we compare the epidemic curves observed for $H^*_{(C=5)}$ and $H^*_{(C=10)}$).

## Results

**i. Under which circumstances may physical distancing increase infection risk?**

**Figure 3:**
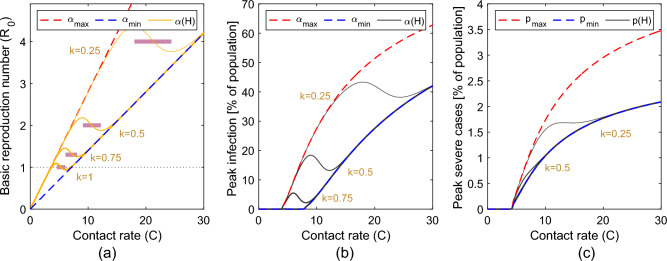
Dynamics occurring when considering that interaction-dependent health benefits acquired through human-to-human contact provide (a,b) infection resistance or (c) disease tolerance. We consider that (a) the basic reproduction number $R_0=C \alpha (H)/\gamma$ (orange curves) and the probability of severe infection *p*(*H*) are directly affected by *H*, and thus indirectly by the contact rate *C*, and so is the proportion of (b) infected and (c) severely infected individuals at the epidemic peak. In (a) and (b) the scattered red and blue lines represent the values of $R_0$ and the corresponding epidemic peaks predicted by a classic SIR model with probability of infection transmission $\alpha _{max}$ or $\alpha _{min}$, respectively. In (c) the scattered red and blue lines represent the proportion of severe cases at the epidemic peak obtained when the probability of experiencing severe illness from infection corresponds to $p_{max}$ and $p_{min}$. The continuous orange and black lines represent results obtained for different *k*, representing the fractional contributions of each contact to an increase in *H* (from thin to thick: $k = 0.25, 0.5, 0.75, 1$). For $k\rightarrow 1$, all contacts contribute to an increase in *H*. For small *k*, only a fraction of these contacts contributes to an increase in *H*.

When the contact rate *C* is small, our SIR+ model follows the behaviour of a classic SIR model with $R_{0_{max}} = C \alpha _{max}/\gamma$ (scattered red lines, Fig. 3a and b). When *C* is large our SIR+ model behaves like a classic SIR model with $R_{0_{min}} = C \alpha _{min}/\gamma$ (scattered blue lines). Thus, for intermediate values of *C* the epidemic dynamics transition from behaving as a disease outbreak characterized by $R_{0_{min}}$, to a disease outbreak characterized by $R_{0_{max}}$, as indicated by the orange and black curves in Fig. 3a and b respectively. The transition may occur smoothly, if the probability of infection transmission decreases linearly as interaction-dependent benefits increase (i.e., if $\alpha (H)$ is modelled as a linear function, see scattered line in Figs. 2a, D.1), or occur in a more abrupt manner, if the probability of infection transmission decreases sharply as soon as a certain threshold of interaction-dependent health benefits is reached (i.e., if $\alpha (H)$ is modelled as a sigmoid function, see solid line in Figs. 2a and 3). Similarly, the scattered red and blue lines in Fig. 3c correspond to the cases for which the probability of severe disease *p* is equal to $p_{max}$ or to $p_{min}$, respectively, while black curves account for a dependency of *p* on *H*.

Increasing the contact rate *C* leads to increased interaction-dependent health benefits and in infection resistance large enough to compensate for the increase in infection transmission (i.e., if for $C_1<C_2$ while $R_{0{(C_1)}} > R_{0{(C_2)}}$, or if $C_1 \alpha _{(H_{(C_1)})} > C_2 \alpha _{(H_{(C_2)})}$), then increasing physical distancing *increases* the basic reproduction number (pink regions in Fig. 3a). This behaviour is counter-intuitive, as it implies that physical distancing may, under certain circumstances, increase the risk of experiencing a larger epidemic outbreak. Similar results are observed when considering that interaction-dependent health benefits affect disease tolerance and the peak in severe cases (Fig. 3c).

Note that a decrease in the probability of infection transmission given a contact from $\alpha _{max}$ to $\alpha _{min}$ implies an increase in the minimal contact rate needed to experiencve an epidemic outbreak. For example, for $\alpha = \alpha _{min} = 0.02$ an outbreak occurs only for $C>7.1$, where $C=7.1$ corresponds to the value for which $R_0 = \alpha _{min} C/\gamma \simeq 1$, while for $\alpha = \alpha _{max}=0.04$ an outbreak occurs for $C>3.6$ (see Fig. 3a). In the extreme case in which interaction-dependent health benefits offer complete resistance against pathogen spread (i.e., $\alpha _{min}=0$), an epidemic outbreak may occur *only* when physical distancing is implemented and a loss in interaction-dependent health benefits is experienced (see Fig. D.2a,b). Similarly, if interaction-dependent health benefits offer full protection against severe illness, severe cases may be observed only when physical distancing is in place (see Fig. D.2c). These situations may occur, for example, if a loss of microbial exposure leads to an increase in the risk of infection and severe illness from diseases that were previously considered innocuous.

**ii. How do differences in the contribution of contact to the acquisition of interaction-dependent health benefits affect the impact of physical distancing?**

Different orange and black curves in Fig. 3 represent model outputs obtained for different values of *k*, that is the fraction of contacts that contributes to an increase in *H*. We observe that when *k* is high (i.e., $k \rightarrow 1$) the range for which increasing the contact rate leads to a decrease in $R_0$ or in a decrease in infection prevalence at the epidemic peak is around lower values of *C*. In these cases, maximizing the number of contacts that provide direct or indirect health benefits may effectively allow for the relaxation of physical distancing, without increasing infection prevalence. If the fraction of contacts that contributes to an increase in *H* is low, however, (i.e., $k \rightarrow 0$), the acquisition of interaction-dependent health benefits impacts infection resistance and disease tolerance only when the contact rate and the expected corresponding epidemic peaks are large.

**iii. What are the possible consequences of the abrupt relaxation of physical distancing on the spread of future epidemics?**

**Figure 4:**
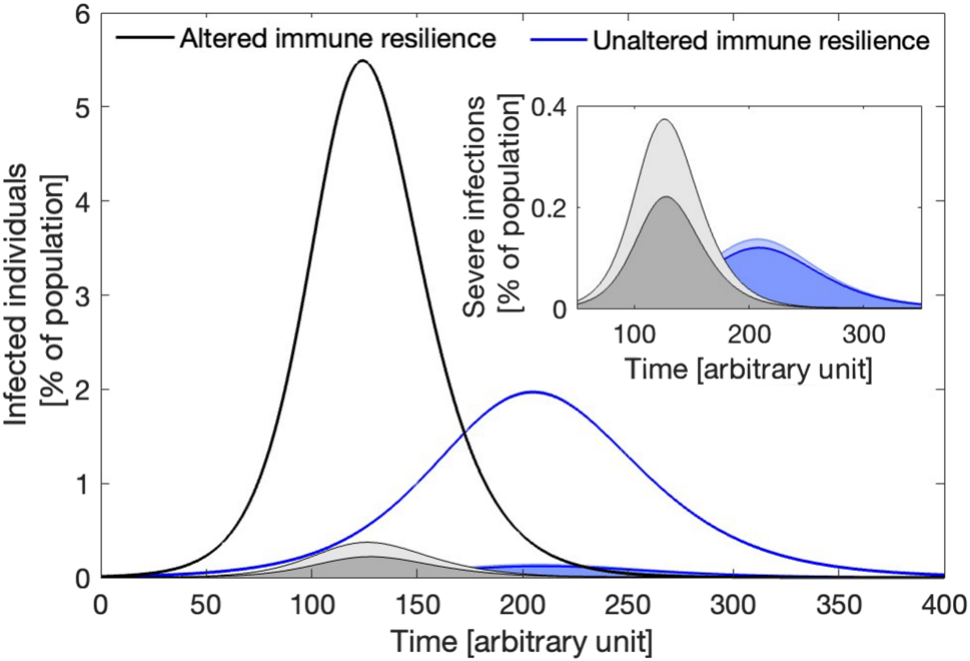
When considering that prolonged physical distancing has long-term consequences on immune resilience, a population experiences stronger and faster epidemics after physical distancing relaxation (black curves) with respect to what would be observed if immune resilience had not been altered (blue curves). In the case in which only a certain percentage of infected individuals suffers from severe illness (shaded areas under the epidemic curves), the fraction of severe cases expected (shaded dark blue area) increases due to a decrease in infection resistance (shaded dark grey area), due to a decrease in disease tolerance (shaded light blue area), or due to both effects (shaded light grey area).

Prolonged periods of physical distancing may lead to changes in lifestyle, altered stress physiology, reduced microbial exposure, and subsequently decreased levels of immune system activation (see section “Processes driving changes interaction-dependent health benefits”). In Fig. 4, we consider that physical distancing measures are relaxed at time zero when only 0.0001 % of a population is infected with a newly introduced disease. We compute the epidemic curves observed when physical distancing has negatively affected a population’s ability to resist infection and tolerate disease (black curves) and compare them with the epidemic curves observed when the population’s ability to resist infection and tolerate disease has not been altered (blue curves). The proportion of individuals experiencing severe effects (shaded dark blue area, Fig. 4) increases in a population with altered immune resistance. This increase can be due to a decrease in disease tolerance, causing an increase in the proportion of people experiencing severe effects (shaded light blue area), to a decrease in infection resistance, and thus an increase in the overall number of infections (shaded dark grey area), or to a combination of these two effects (shaded light grey area). The feasibility of such scenarios depends on the time required for a population to reacquire the immune defences lost during the isolation period (i.e., on the ratio $h/\delta$ in our model, which determines how fast a population recovers from a loss in interaction-dependent health benefits, see section C).

## Discussion

**i. Under which circumstances may physical distancing increase infection risk?**

Physical distancing measures are key for infectious disease control, and overall distancing will have mostly and overwhelmingly positive effects on preventing infectious spread. However, its consequences extend far beyond reduced pathogen transmission, and thus the impact of physical distancing on a population’s health may not be strictly positive. Although in classical compartmental models increasing physical distancing always corresponds to a reduction in infections and hospitalizations, in reality the impact of physical distancing on disease susceptibility is multifaceted, and not always easy to unveil. Multiple psychological, social, and medical studies conducted during and after the implementation of COVID-19 public health interventions clarify that physical distancing measures impeded several dimensions of our psychosocial well-being and health^40,129,130^. These studies are key to the development of novel quantitative approaches that account for positive as well as negative consequences of physical distancing on the infection dynamics, and our SIR+ model is an example of how epidemiological models may incorporate these considerations.

If we recognize that physical distancing has a cost, such as increased stress physiology or decreased exposure to microbiota diversity, and if this cost affects our ability to resist and tolerate infections, then increasing physical distancing may not necessarily mean improved population health. Indeed, if physical distancing creates a situation in which people are less fit, eat less healthily, are more stressed, or feel more lonely, and if these changes compromise our immune functioning, affect our susceptibility to infections or aggravate disease severity^49–53^, physical distancing implementation may, under certain circumstances, increase outbreak severity, or create the emergence of medical issues that were absent at first.

An important implication of physical distancing on health is that its effect can create even more dramatic dynamics than the model predicts. For instance, not only can social isolation affect immunity through the microbiome and stress hormone system, but the microbiome and stress physiology also both affect each other and social behaviour^47,72–75,131^. This interdependence creates potential for feedback loops whereby, for example, social isolation leads to a depleted microbiome and altered stress physiology (through the hyper-or hyporeactive hypothalamic-pituitary-adrenal (HPA) axis) and these two phenomena can strengthen each other (since stress is known to lower microbiome diversity, and low microbiome diversity is known to sensitize hosts to stressors), where both higher stress and depleted microbiome diversity can make the host less sociable, and thus more socially isolated^132,133^.

The link between increased physical distancing and increased disease susceptibility is strongly dependent on multiple factors, such as the inherent properties of the pathogen and our microbiome and immune system and, when considering psychological well-being, on a population’s behavioural response to public health policies^134,135^. The basic reproduction number $R_0$ can be considered the average number of infected contacts per individual in a population. Similarly, *H* is also a population-averaged value representing how contact affects the acquisition and loss of health benefits in a population. In reality, however, infection transmission rates and health benefits acquisition differ in sub-populations with different age structure, distribution of co-morbidities, or spatial structure and connectivity. For instance, children and youth may be more likely to suffer from microbiome depletion and loneliness during social isolation, and groups facing economic insecurity may experience an increased risk to suffer from anxiety and depression^17,18,36,48^. Future modelling may consider how populations heterogeneity affects the acquisition and loss of interaction-dependent health benefits, in addition to affecting the infection dynamics^57,58^.

Differences between individuals can also lead to changes in the dynamics of infection. When considering compartmental models, super-spreading events in which the minority of individuals infect disproportionately more susceptible contacts (as compared to most individuals infecting few or no others) may change the expected epidemiological outcome^136^. An aspect of this phenomenon that is particular to the SIR+ model may occur in the presence of a set of individuals with disproportionate influence on the acquisition of interaction-dependent health benefits. For example medical clowns, thank to their positive role in building social connections, dealing with emotions, caring and encouragement, and motivating treatment adherence^137^, can have a particular impact on *H* and act as ‘super-spreaders of health’. In more traditional societies, Shamans and traditional healers may have a similar role^138^. Moving forward, experimental and theoretical work will be crucial for the quantification and incorporation of multiple indicators of health and their interplay in epidemiological models, which would allow us to transition toward the management of future epidemics that includes producing guidelines based on a broader definition of health and infection risk.

**ii. How do differences in the contribution of contact to the acquisition of interaction-dependent health benefits affect the impact of physical distancing?**

Seminal work in the past decade highlighted the difference between cleanliness (i.e., the mere absence of dirt) and hygiene (i.e. what we do to protect against infection) to develop and promote a ‘targeted hygiene’ approach; namely guidelines on how to encourage hygiene without necessarily reducing our exposure to essential microbes^139^. Other work has discussed how understanding the relative importance of different types of social contact for parasite transmission may contribute to improved infectious disease control^87^. For example, considerations on how to eliminate pathogens from critical central points should be accompanied by recommendations on which types of interactions should we have to promote exposure to essential microbes, whose acquisition may affect the average susceptibility to infection and the incidence of severe disease.

Our SIR+ model takes these considerations into account by distinguishing between the contact rate (parameter *C* in the model), the probability of infection given a contact ($\alpha$), and the fraction of contacts that contributes to the acquisition of health benefits (*k*). Thus although both pathogenic spread and interaction-dependent benefits increase through contact, a balance between these two processes can be achieved through the implementation of interventions aimed at minimizing $\alpha$ while maximizing *k*. The classical example is the use of masks, which helps reduce the spread of airborne diseases, while simultaneously allowing social gatherings and exposure to other essential microbial agents (i.e., reduce the probability of infection given a contact, $\alpha$ while leaving *k* unaffected, or only slightly reduced).

Other strategies aimed to reduce social isolation and improve psychological well-being while minimizing pathogen spread can also be crucial for our health during a pandemic^48,140,141^, where outcomes may vary as a function of the nature of the interactions. For example, one might expect high infection transmission when touching a loved one compared to chatting with them, and Spencer et al.^142^ discussed how the infection risk due to spontaneous face and eye-touching may be accompanied by a gain in mutualistic microbiota. The interaction type may also impact stress levels and mental health. Adolescents who reported more social connectedness pre-COVID-19 had lower depression symptoms and higher mental health one year post COVID-19, compared to adolescents reporting lower social connectedness, suggesting that engaging with close ones and establishing a sense of community may serve as protective factors amid physical distancing^143^. A recent review suggests that social isolation is associated with worse psychological well-being, supporting the notion that interactions that maintain or support relationships with close ones may be especially useful in reducing stress, thereby reducing disease susceptibility^144^.

Besides the implementation of specific targeted interventions, contact network structure can also have a strong influence on infection transmission dynamics. Multiple theoretical approaches have been developed to determine how infectious disease control may be achieved through targeted network manipulation^145,146^. For example, removing the small number of highly connected individuals in a contact network has proven to be a very effective strategy for controlling infectious disease spread in a population^147,148^. Increasing network clustering would also slow down epidemic spread, due to the rapid depletion of local susceptible contacts^149,150^. Future work may consider how policies may be designed such that they would lead to the emergence of social networks of interaction that minimize pathogen transmission (and thus minimize the probability of infection given a contact $\alpha$) but also to maximize other interaction-dependent factors that may affect our health and well-being (and thus maximize the fraction of contacts that leads to health benefits *k*). Experiments on wild mice, for example, have shown that mice in bridge-type central positions (e.g., contact with high betweenness, or high bridge propensity), present higher microbiome diversity with respect to other mice^66^. However, measures of general centrality (such as eigenvector centrality) are important determinants of pathogen transmission and host probability of infection^151,152^, which suggests that changes in a network structure may differently impact pathogen transmission (i.e., $\alpha$) and the transmission of beneficial microbes (i.e., *k*). Complementing network studies on how to minimize pathogen transmission with recommendations on how to maximize other health benefits may be key to ensuring that implemented policies truly benefit health and that their potential impact in slowing down infectious disease spread is maximized.

**iii. What are the possible consequences of the abrupt relaxation of physical distancing on the spread of future epidemics?**

When COVID-19 restrictions were eased, different countries in the world experienced out-of-season epidemics of respiratory syncytial virus (RSV) and influenza, especially among young children^153–157^. These epidemics were characterized by unexpected timing and severity, and have caused a shortage in pharmaceutical products due to pediatric hospitals and pharmacies’ unpreparedness^158–161^. Although mechanisms leading to this out-of-season outbreak are not clearly understood, their link to the COVID-19 pandemic is indisputable. Possible reasons for the resurgence of strong epidemics have been attributed to decreased population immunity following a prolonged period of minimal exposure to pathogenic agents (also referred to as ‘immune debt’ or ‘immunity gap’)^19,26,162–165^. Indeed, low exposure to viruses with transient immunity (such as RSV) may affect a whole population’s susceptibility to infection and lead to increased community transmission, where children, the elderly, and immuno-compromised individuals are particularly vulnerable to infection^26,164^.

Here we raise the possibility that a population that experienced prolonged periods of physical distancing can also suffer from increased susceptibility to infection due to increased psychological stress or to reduced microbial exposure, caused by a reduction in human-to-human interaction and microbial sharing^166^. In this respect, a gradual relaxation of physical distancing measures may be important to ensure that the population (and in particular vulnerable individuals) will have time to replenish a healthy microbiome and re-acquire normal levels of immune activation and colonization resistance. Gradual relaxation may be especially important if the population has experienced an epidemic wave while physical distancing measures were in place, which may have driven a further decrease in microbial exposure, leading to both more susceptible individuals within the population as well as a longer time required by a population to reacquire the health benefits lost during social isolation. Our SIR+ takes this latter scenario into account by considering that microbiome depletion leads to a reduction in the rate at which interaction-dependent benefits are acquired in a population, by affecting the extent to which interaction with infected and recovered individuals contributes to an increase in *H* (i.e., parameters $p_I$ and $p_R$, see Eq. 1a).

An alternative to gradual relaxation, could be the implementation of short periods of strict physical distancing (i.e., ‘precautionary breaks’, or ‘circuit breaks’;^167–169^), followed by periods in which public health measures are released. This approach may allow for both a decrease in infection prevalence during isolation periods and the accumulation of interaction-dependent health benefits when physical distancing measures are lifted. The optimal duration and intensity of the precautionary breaks may be evaluated based on the rate at which interaction-dependent health benefits are lost during social isolation, beside accounting for other epidemiological and economic indicators.

## Concluding remarks

The common perception derived from classic SIR models is that the rate of infection transmission is proportional to the strength of physical distancing. However, this understanding does not take into account potential negative feedback occurring when the contact rate is small. Indeed, a strong reduction in human-to-human contact may increase anxiety and depression in a population, or cause a lack of interactions of importance with a wide variety of microbes, thereby affecting a population’s ability to resist infection and tolerate disease and, consequently, affect infection risk in a non-intuitive manner. Our SIR+ model presents a possible way to quantify this feedback, and overall contributes to creating more awareness about non-linear effects in the evaluation of infection risk associated with the implementation of prolonged physical distancing. Ultimately, this can help us develop new ways of modelling dynamics of health and disease, whereby we update our historical ideas of health as an individual trait and pathogens as elements that spread among individuals, and consider also aspects of health that can spread between people and pathogenesis as something that arises context-dependently.

### Supplementary Information

Supplementary Information.

## Data Availability

The datasets generated or analyzed during the study are available in this published article. All the code developed for the manuscript is available on GitHub. The code is novel and original, and has been developed by author Maria M. Martignoni (2023) and can be found at https://github.com/nanomaria/SIRplus.
