# Supplementary material for: SIR+ models: accounting for interaction-dependent disease susceptibility in the planning of public health interventions
Source: Sci Rep. 2024 Jun 5;14:12908. doi: 10.1038/s41598-024-63008-9 (PMC11153654; doi:10.1038/s41598-024-63008-9)
Supplement: Supplementary file 1 — Supplementary Information. [file 41598_2024_63008_MOESM1_ESM.pdf]

Supplementary information

SIR+ models: Accounting for interaction-dependent disease susceptibility in the planning of public health interventions

Maria M. Martignoni, Aura Raulo, Omer Linkovski, Oren Kolodny

Contents

1 Introduction **3**

2 Model and Methods **4**

2.1 The SIR+ model 4
2.2 Processes driving changes interaction-dependent health benefits 5
2.2.1 Acquisition of interaction-dependent health benefits 5
2.2.2 Loss of interaction-dependent health benefits 7
2.3 Processes determining infection transmission and disease severity 8
2.3.1 Infection resistance and the $\alpha(H)$ function 8
2.3.2 Disease tolerance and the $p(H)$ function 9
2.4 The role of the contact rate C and scenarios of interest 10

3 Results **11**

4 Discussion **13**

5 Concluding remarks **17**

A Default parameters **32**

B Function $p(H)$ **33**

C Acquisition and loss of interaction-dependent health benefits over time **33**

D Results obtained for linear $\alpha(H)$ and $p(H)$, and for $\alpha_{min}, p_{min} = 0$ **36**

A Default parameters

Table A.1: Variables and default parameter values chosen for the simulations.

Symbol	Description	Unit	Default value
S	Proportion of susceptible population	–	–
I	Proportion of infected population	–	–
R	Proportion of recovered population	–	–
H	Average interaction-dependent health benefits of a population	Health benefits	–
C	Contact rate	Contact/time	0-30
k	Fractional contribution of each contact to an increase in H	–	0.25, 0.5, 0.75, 1
h	Maximal interaction-dependent health benefits acquired from each contact	Health benefits/contact	0.1
γ	Recovery rate	1/time	1/7
η	Recovery rate (severe cases)	1/time	1/10
δ	Rate of loss of interaction-dependent benefit	1/time	0.1
α_{max}	Probability of infection transmission given a contact (low H)	1/contact	0.04
α_{min}	Probability of infection transmission given a contact (high H)	1/contact	0.02
p_{max}	Probability of severe illness from infection (low H)	–	blue 0.05
p_{min}	Probability of severe illness from infection (high H)	–	0.03
p_I	Adjusted contribution of infected individuals to the acquisition of interaction-dependent health benefits	–	0.6
p_R	Adjusted contribution of recovered individuals to the acquisition of interaction-dependent health benefits	–	0.8
H_{max}	Critical value of H above which interaction-dependent health benefits can no longer be acquired through contact	Health benefits	10
H_i^α	Critical value of H determining a change in infection resistance	Health benefits	5
H_i^p	Critical value of H determining a change of disease tolerance	Health benefits	5

B Function $p(H)$

Function $p(H)$ represents the dependency on H of the probability p of experiencing severe illness from infection, and can be modelled equivalently to the probability of infection transmission $\alpha(H)$ (see Eqs. 3 and Eqs. 4, in the main manuscript). When considering that p assumes a sigmoid form, we write

$$p(H) = p_{min} + \frac{p_{max} - p_{min}}{2}[1 - \tanh((H - H_i^p)] \,. \tag{6}$$

Alternatively, the function $p(H)$ may assume a linear form, as

$$p(H) = \begin{cases} -\dfrac{(p_{max} - p_{min})}{2\,H_i^p} H + p_{max} & \text{for} \quad H \leq 2\,H_i^p \,, \\ p_{min} & \text{for} \quad H > 2\,H_i^p \,. \end{cases} \tag{7}$$

The graphical representation of Eqs. (6) and (7) is provided in Fig. 2(b).

C Acquisition and loss of interaction-dependent health benefits over time

Consider changes in the average interaction-dependent health benefits H experienced by an initially susceptible population over time. From Eqs. (1) and (2), we know that:

$$\frac{dH}{dt} = \begin{cases} C\,k\,h\, - \delta H & \text{for} \quad \mathrm{H} \leq \mathrm{H_{max}} \,, \\ \quad -\delta H & \text{for} \quad \mathrm{H} > \mathrm{H_{max}} \,. \end{cases} \tag{8}$$

Note that, for constant contact rate C and in a fully susceptible population (i.e., when $S = 1$ and $I, R = 0$), H reaches an equilibrium H^* which corresponds to

$$\lim_{t \to \infty} H(t) = H^* = \begin{cases} C\,k\,\frac{h}{\delta} & \text{for} \quad C\,k\,\frac{h}{\delta} \leq H_{max} \,, \\ H_{max} & \text{for} \quad C\,k\,\frac{h}{\delta} > H_{max} \,. \end{cases} \tag{9}$$

As Eq. (8) is not continuous, in order to solve it numerically, we use a smooth version of the function dH/dt, for which $\zeta(H)$ is given by

$$\zeta(H) = C\,k\,h\,\frac{1}{2}[1 - \tanh(3(H - H_{max}))] \tag{10}$$

and the differential equation dH/dt is given by

$$\frac{dH}{dt} = C\,k\,h\,\frac{1}{2}[1 - \tanh(3(H - H_{max}))] - \delta H \,. \tag{11}$$

A graphical representation of Eq. (10) is provided in Fig. C.1.

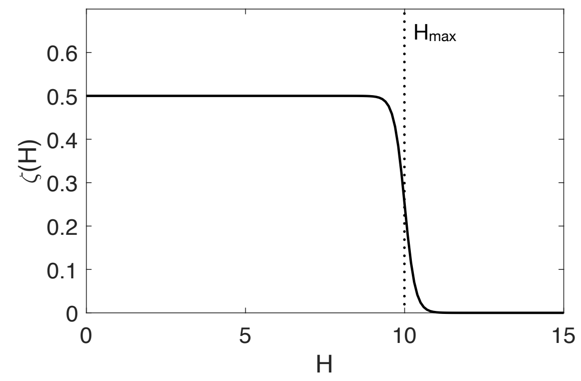

Fig. C.1: Function $\zeta(H)$ of Eq. (10), with $H_{max} = 10$, $C = 10$, $h = 0.1$ and $k = 0.5$.

In Fig. C.2, the solution $H(t)$ of Eq. 11 is plot as a function of time for different contact rates (parameter C) and for different fractional contribution of each contact to H (parameter k). As given in Eq. (9), the solution $H(t)$ tends to the equilibrium value $H^* = Ckh/\delta$ for $H \leq H_{max}$. When $H > H_c^m$, the value of $H(t)$ would decrease and stabilize around $H^* = H_{max}$. Note that, for constant ratio h/δ, the equilibrium value H^* is reached much faster for a higher contact rates C and when a higher fraction of contacts contributes to the acquisition of health benefits (parameter k). Increasing the ratio h/δ will also cause the equilibrium value to be reached faster.

In Figs. C.3 and C.4 we show how interaction-dependent health benefits (H), the probability of infection transmission given a contact ($\alpha(H)$) and the probability of experiencing severe disease ($p(H)$) vary as a function of the contact rate C, in an initially fully susceptible population.

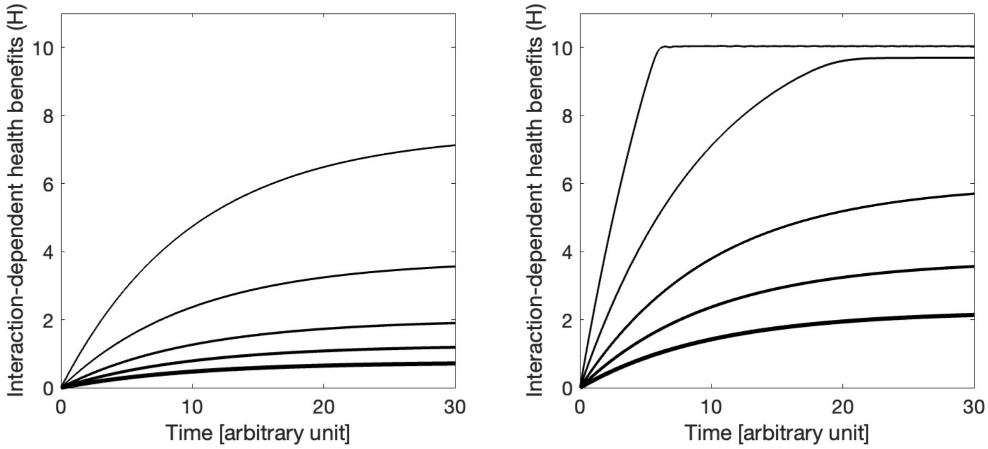

Fig. C.2: The average interaction-dependent health benefits H experienced by a population as a function of time, for different contact rates (from the thicker to thinner lines: $C = 3, 5, 8, 15, 30$, for (a) $k = 0.25$, and (b) $k = 0.75$. For the simulations, dH/dt is modeled according to Eq. 11, with $H_{max} = 10$

. Other default parameter values are provided in Table A.1.

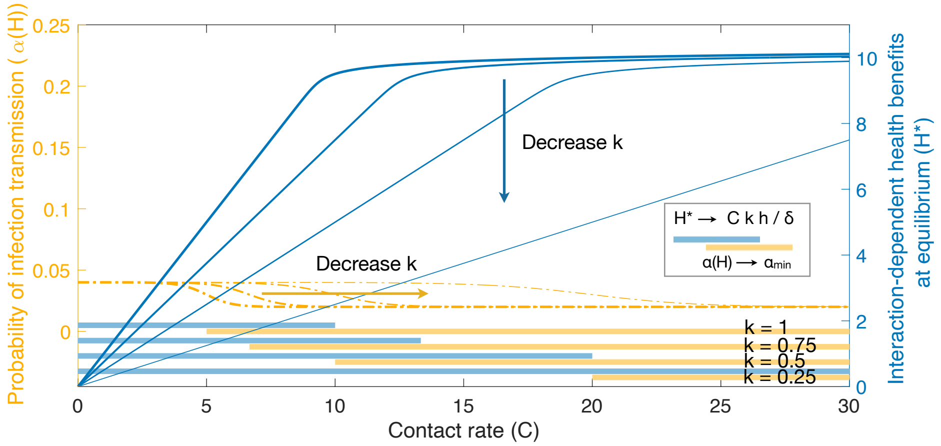

Fig. C.3: Left vertical axis: Probability of infection transmission given a contact (i.e., function $\alpha(H)$, Eq. (4)), as a function of the contact rate C. An equivalent plot can be obtained when considering disease tolerance, and the probability of experiencing severe illness from infection (i.e., function $p(H)$, see Fig. C.4). Right vertical axis: Average interaction-dependent health benefits H experienced by a population at equilibrium (H^*, Eq. (9)), as a function of the contact rate C. Both functions are represented for $k = 0.25, 0.5, 0.75, 1$, with $k = 1$ indicating that all contacts contribute to an increase in H, and $k = 0.25$ indicating that only 1/4 of the contacts contribute to an increase in H. Note that $H^* = Ckh/\delta$ for $Ckh/\delta \leq H_{max}$, and $H^* = H_{max}$ for $Ckh/\delta > H_{max}$ (see Eq. 9). The parameter range of C for which $H^* \to Ckh/\delta$ (i.e., $C = [0, H_{max}\delta/(hk)]$) for different values of k is indicated in blue at the bottom of the figure. The probability of infection given a contact $\alpha(H)$ decreases with increasing H^*, and thus with increasing C, where $\alpha(H)$ tends to its minimal value α_{min} when $H > H_i^\alpha$. The range $C > H_i^\alpha \delta/k$ for which $\alpha(H) \to \alpha_{min}$ is indicated for different values of k in orange at the bottom of the figure. Default parameter values chosen for the simulations are provided in Table A.1.

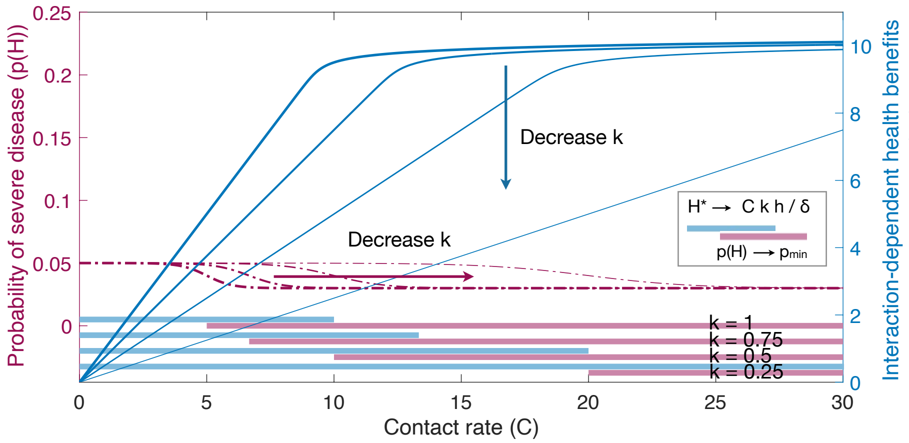

Fig. C.4: Left vertical axis: Plot of disease tolerance, expressed as the probability of experiencing severe illness from infection (i.e., function $p(H)$, Eq. 6), as a function of the contact rate C. Right vertical axis: Average interaction-dependent benefits H experienced by a population at equilibrium (i.e., H^*, Eq. (9)), as a function of the contact rate C. Both functions are represented for $k = 0.25, 0.5, 0.75, 1$. See Fig. C.3 for a more detailed explanation of the figure.

D Results obtained for linear $\alpha(H)$ and $p(H)$, and for $\alpha_{min}, p_{min} = 0$

We would like to explore how the scenarios described in Fig. 3 may change when considering linear $\alpha(H)$ and $p(H)$ functions (Eqs. (3) and (7)), instead of a sigmoid function (Eqs. (4) and (6)). Additionally, we investigate the impact of setting parameters α_{min} and p_{min} to zero on the dynamics. Results are shown in Figs. D.1 and D.2.

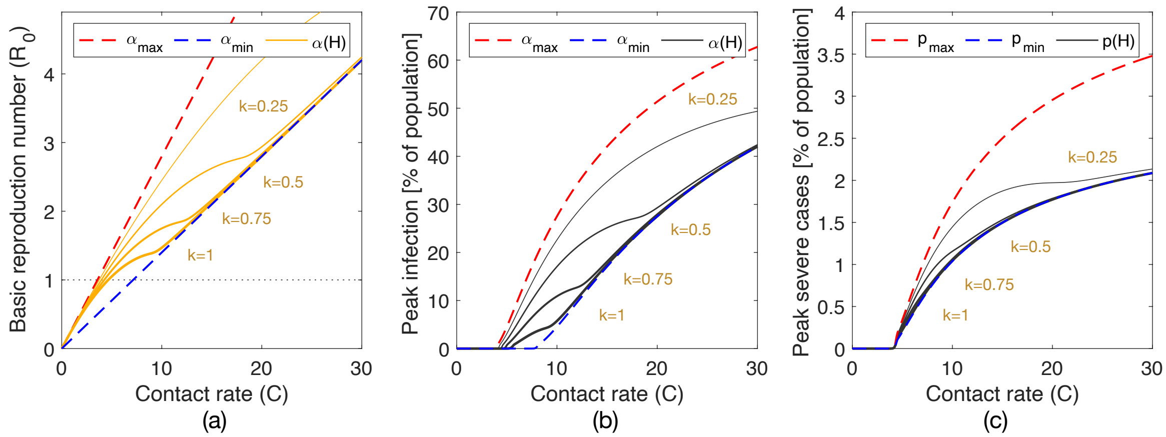

Fig. D.1: Plot equivalent to Fig. 3, but considering that (a,b) the probability of infection transmission given a contact ($\alpha(H)$), and (c) the probability of experiencing severe illness ($p(H)$), decrease linearly with increasing interaction-dependent health benefits (see Eqs. (3) and (7)).

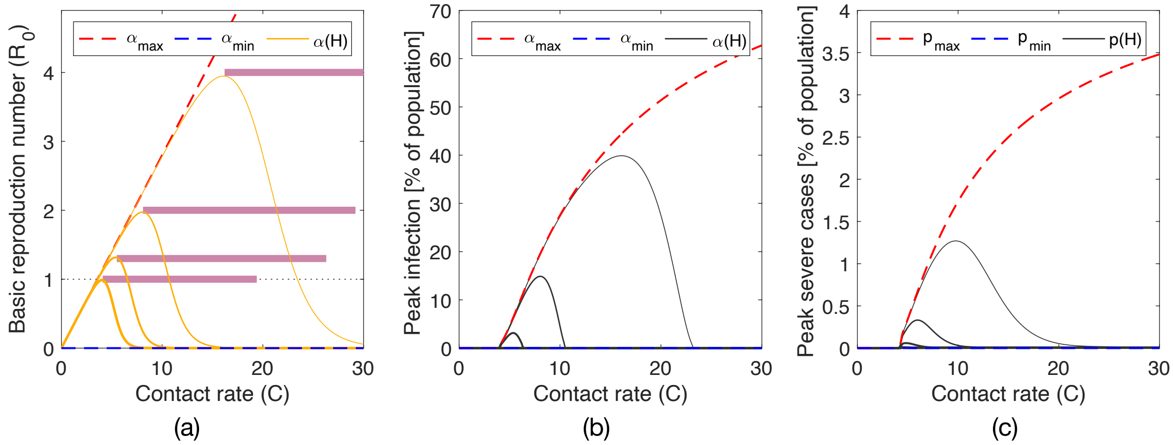

Fig. D.2: Plot equivalent to Fig. 3, but considering that (a,b) the probability of infection transmission given a contact α_{min} and (c) the probability of experiencing severe illness p_{min} reduce to zero when interaction-dependent health benefits are high. Note that in this case (b) an epidemic outbreak, or (c) severe disease occurs only if physical distancing is in place.
